# P07-11 Physical activity promotion in the German healthcare system - Establishing pathways of exercise referral for persons with noncommunicable diseases

**DOI:** 10.1093/eurpub/ckac095.111

**Published:** 2022-08-29

**Authors:** Sarah Klamroth, Inga Naber, Eriselda Mino, Wolfgang Geidl, Peter Gelius, Karim Abu-Omar, Klaus Pfeifer

**Affiliations:** Department of Sport Science and Sport, Friedrich-Alexander-University Erlangen-Nürnberg, Erlangen, Germany

**Keywords:** exercise referral, physical activity promotion, primary healthcare, noncommunicable diseases

## Abstract

**Background:**

Exercise referral schemes (ERS) are embedded in the routine practice of healthcare systems in many countries (e.g. Sweden and New Zealand). In primary healthcare, ERS are recommended to sustainably increase physical activity (PA) levels among patients with noncommunicable diseases (NCD). Yet, the German health care system currently only incorporates interventions that primarily focus on improving functional outcomes but hardly aim at increasing PA levels. This presentation introduces an ongoing research project that aims to develop, implement and evaluate an ERS to promote PA for persons with NCD within the German healthcare system.

**Methods:**

In the first phase of the project, a concept of an ERS was developed using a participatory research approach that involved relevant stakeholders such as physicians, funding agencies, PA providers, and patient representatives. The development process comprised three steps: 1) interviews to gather stakeholders' ideas of an ERS; 2) a literature review to collect evidence on key elements of international ERS; 3) three stakeholder meetings to combine scientific evidence with stakeholders' perspectives (co-creation). Subsequently, the ERS will be implemented, tested and evaluated in a regional pilot project using a pragmatic trial design. Finally, a concept for scaling-up the ERS to the German national level will be developed.

**Results:**

As result of the co-creation process, the following key elements were defined to be part of the ERS: Screening, short counselling and provision of a referral form by a physician; initial assessment, counselling, individual PA recommendations, re-assessment and follow-up by exercise professionals. Additional aspects considered important for the implementation of the ERS were ensuring good communication and feedback between all participating health professionals, as well as an overview of all local physical activity offers and exercise professionals (database). These preliminary findings were combined into a draft of the ERS.

**Conclusions:**

The participatory research approach employed by our project yielded the first draft of an ERS with a specific focus on PA promotion among persons with NCD within the German healthcare system. In the upcoming project stage, this ERS concept will be further elaborated and prepared for implementation and evaluation at a regional level.

